# Biochemical and transcriptomic analyses reveal different metabolite biosynthesis profiles among three color and developmental stages in ‘Anji Baicha’ (*Camellia sinensis*)

**DOI:** 10.1186/s12870-016-0885-2

**Published:** 2016-09-08

**Authors:** Chun-Fang Li, Yan-Xia Xu, Jian-Qiang Ma, Ji-Qiang Jin, Dan-Juan Huang, Ming-Zhe Yao, Chun-Lei Ma, Liang Chen

**Affiliations:** 1 Key Laboratory of Tea Biology and Resources Utilization, Ministry of Agriculture, Tea Research Institute of the Chinese Academy of Agricultural Sciences, Hangzhou, China; 2 School of Agriculture and Food Science, Zhejiang Agriculture and Forestry University, Lin’an, Hangzhou China

**Keywords:** Albino, *Camellia sinensis*, Carotenoids, Chlorophylls, RNA-seq, Theanine, Tea plant

## Abstract

**Background:**

The new shoots of the albino tea cultivar ‘Anji Baicha’ are yellow or white at low temperatures and turn green as the environmental temperatures increase during the early spring. ‘Anji Baicha’ metabolite profiles exhibit considerable variability over three color and developmental stages, especially regarding the carotenoid, chlorophyll, and theanine concentrations. Previous studies focused on physiological characteristics, gene expression differences, and variations in metabolite abundances in albino tea plant leaves at specific growth stages. However, the molecular mechanisms regulating metabolite biosynthesis in various color and developmental stages in albino tea leaves have not been fully characterized.

**Results:**

We used RNA-sequencing to analyze ‘Anji Baicha’ leaves at the yellow-green, albescent, and re-greening stages. The leaf transcriptomes differed considerably among the three stages. Functional classifications based on Gene Ontology enrichment and Kyoto Encyclopedia of Genes and Genomes enrichment analyses revealed that differentially expressed unigenes were mainly related to metabolic pathways, biosynthesis of secondary metabolites, phenylpropanoid biosynthesis, and carbon fixation in photosynthetic organisms. Chemical analyses revealed higher β-carotene and theanine levels, but lower chlorophyll *a* levels, in the albescent stage than in the green stage. Furthermore, unigenes involved in carotenoid, chlorophyll, and theanine biosyntheses were identified, and the expression patterns of the differentially expressed unigenes in these biosynthesis pathways were characterized. Through co-expression analyses, we identified the key genes in these pathways. These genes may be responsible for the metabolite biosynthesis differences among the different leaf color and developmental stages of ‘Anji Baicha’ tea plants.

**Conclusions:**

Our study presents the results of transcriptomic and biochemical analyses of ‘Anji Baicha’ tea plants at various stages. The distinct transcriptome profiles for each color and developmental stage enabled us to identify changes to biosynthesis pathways and revealed the contributions of such variations to the albino phenotype of tea plants. Furthermore, comparisons of the transcriptomes and related metabolites helped clarify the molecular regulatory mechanisms underlying the secondary metabolic pathways in different stages.

**Electronic supplementary material:**

The online version of this article (doi:10.1186/s12870-016-0885-2) contains supplementary material, which is available to authorized users.

## Background

The tea plant [*Camellia sinensis* (L.) O. Kuntze] is cultivated worldwide for the production of nonalcoholic beverages. Most tea plants have normal, green leaves. Tea plant breeders have developed several cultivars with distinct shoot colors (e.g., yellow, white, and purple), which have become valuable materials for the production of unique green teas with specific colors and flavors. In Anji County of southwestern China, as well as in nearby regions, several cultivars (e.g., ‘Anji Baicha’) that produce white leaves are cultivated. ‘Anji Baicha’ is a green-revertible albino tea cultivar that produces yellow or white shoots at low temperatures. An increase in temperature turns the shoots green [1]. Inhibiting chloroplast development and chlorophyll accumulation leads to the yellow or white shoots observed during the albescent stages. With increasing temperatures, the chloroplast structure recovers and chlorophyll contents increase, causing the leaves to become green [1–4]. Investigations of another yellow-leaf tea cultivar, ‘Zhonghuang 2’, revealed that the chloroplast ultrastructure is disrupted, with poorly stacked grana and lower chlorophyll *a* and *b* contents than in the green tea cultivar ‘Longjing 43’ [5].

Several methods have been applied to investigate the mechanism mediating the albino phenotype in ‘Anji Baicha’ plants. Using microarrays, differentially expressed genes (DEGs) were detected in various albescent stages. Most of these genes are related to chlorophyll and protein biosynthesis [6]. Analyses focused on amplified fragment length polymorphisms have helped identify DEGs during periodic albinism, including transcription factor genes as well as genes related to ubiquitination, chloroplast biogenesis, signal transduction, stress responses, cell cycles, and carbohydrate and energy metabolism [7]. A proteome-level analysis of young leaves at three developmental stages revealed that differentially expressed proteins are mainly involved in activities related to photosynthesis, protein processing, and the metabolism of carbon, nitrogen, and sulfur [3].

Changes to leaf color in ‘Anji Baicha’ new shoots during the early spring are accompanied by alterations in metabolite profiles. The metabolites whose production differs between stages are primarily related to carbon fixation in photosynthetic organisms and phenylpropanoid and flavonoid biosyntheses. The carbohydrate and amino acid metabolic pathways are the main disturbed pathways during the albescent stages than during the green stage [8]. Additionally, the abundance of metabolites related to the health effects and sensory qualities of tea varies between developmental stages. During the albescent stage, ‘Anji Baicha’ leaves contain high concentrations of free amino acids, especially theanine [9], which is a unique free amino acid that accounts for approximately 50 % of the total free amino acid content in tea. It gives tea a unique taste known as “umami” [10]. Very little is currently known about the molecular mechanisms regulating theanine biosynthesis during the albescent stage of ‘Anji Baicha’ tea plants.

The low chlorophyll contents of albescent stage ‘Anji Baicha’ leaves are responsible for the production of unique leaf colors. However, the molecular mechanisms responsible for metabolizing chlorophyll in ‘Anji Baicha’ leaves during the albescent stage have not been fully characterized. In higher plants, carotenoids perform key functions related to light harvesting and protection against the effects of excessive light [11]. Tea cultivars with high carotenoid levels produce flavorful teas. During the production of tea, carotenoids are degraded to many flavored volatile compounds that are closely related to tea quality [12]. Previous studies concluded that the total carotenoid content is lower in ‘Anji Baicha’ plants during the albescent stage and in chlorina tea plants than in normal green tea cultivars [5, 9]. However, the molecular mechanisms influencing carotenoid biosynthesis throughout the color and developmental stages of ‘Anji Baicha’ tea plants have not been clarified. To resolve these issues, we collected leaf samples from the yellow-green, albescent, and re-greening stages. First, we characterized the global gene expression patterns for each color and developmental stage via RNA-sequencing (RNA-seq). Based on these results, we assembled a complete gene set, including the genes expressed in different stages. Second, we identified large sets of unigenes differentially expressed between stages. Furthermore, the concentrations of carotenoids, chlorophylls, and theanine in the different stages were analyzed, and the expression patterns of key genes encoding enzymes involved in the metabolism of these compounds were characterized. Finally, analyses of the correlation among expression of unigenes and metabolites concentrations were used to identify the key genes regulating the differential biosynthesis of the metabolites associated with carotenoid, chlorophyll, and theanine biosynthetic pathways.

Our results revealed the dynamic regulation of metabolite biosynthesis in ‘Anji Baicha’ leaves during the various color and developmental stages. We have clarified the regulation of gene expression in ‘Anji Baicha’ leaves during different color and developmental stages, which may be relevant for tea breeding and germplasm improvement.

## Results and discussion

### Color changes and development of ‘Anji Baicha’ leaves in the early spring

**Fig. 1:**
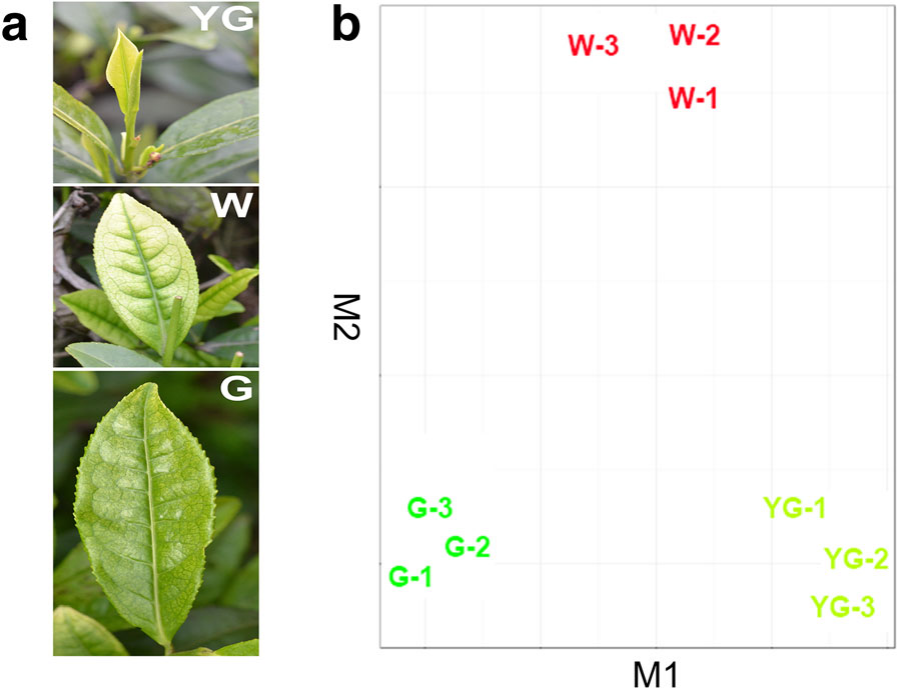
‘Anji Baicha’ tea leaves and transcriptome profiles in different stages. **a** YG, yellow-green leaf; W, white leaf; G, re-greening leaf. Details for each tissue are described in the Methods section under “Plant materials”. **b** CummeRbund was used to analyze the leaf transcript level data from the three analyzed stages (three biological replicates). The RNA-sequencing samples clustered into three groups of three replicate samples according to these stages. These results indicated that the three biological replicates produced consistent gene expression data at each stage, and that the expression levels of unigenes in leaves differed among stages

In early spring, the color of ‘Anji Baicha’ new shoots is affected by environmental temperatures. At temperatures below 20 °C, new shoots are yellow-green or white. With increasing temperatures, new shoots gradually turn green. The albino phenotype is closely related to chlorophyll synthesis, which is inhibited at low temperatures and restored when the temperature increases [2]. The development of new shoots was divided into three stages based on differences in leaf color (Fig. 1a). The initial germination period, in which plants consisted of one yellow-green leaf and one bud, was defined as the yellow-green (YG) stage. During this stage, leaves were light green with a yellow edge. As the first leaf developed, it became off-white and only the leaf vein remained green. This period was defined as the albescent (W) stage. When temperatures increased above 22 °C, leaves grew larger and gradually turned green, similar to the leaves of other tea cultivars. This stage was defined as the re-greening (G) stage.

### RNA-sequencing, unigene assembly, and annotation of the ‘Anji Baicha’ transcriptome

To analyze the leaf transcriptomes at the YG, W, and G stages, nine cDNA libraries (i.e., each stage in triplicate) were created and sequenced using the Illumina HiSeq 2000 platform. The total number of raw reads for the nine samples was 29–48 million (Table 1). Additionally, only one sample contained fewer than 30 million reads. In total, 329 million short reads were generated for the three different stages, and 328 million high-quality 100-bp reads were selected for further analysis.

**Table 1:** Overview of the sequencing and assembly of the ‘Anji Baicha’ transcriptome

	No. of reads	No. of bases (bp)
Yellow-Green-1	42,522,652	6,314,426,906
Yellow-Green −2	48,556,376	7,183,288,820
Yellow-Green −3	32,069,150	4,767,162,763
White-1	31,243,978	4,652,691,537
White-2	37,897,020	5,632,403,468
White-3	37,230,292	5,571,332,617
Green-1	38,222,704	5,693,731,119
Green-2	29,516,642	4,409,674,597
Green-3	31,963,864	4,665,175,947
Total raw data	329,222,678	48,889,887,774
Total high-quality data	328,386,010	48,553,465,996
Average high-quality read length (bp)	147.9	
Average unigene length (bp)	987.2	
Range of unigene length (bp)	201–28,934	
Unigenes ≥200 bp	179,951	177,648,352
N50 (bp)	1,648	

Based on the high-quality reads, the unigenes were assembled using Trinity. Ultimately, we obtained 179,951 unigenes, with average and total lengths of 987 bp and 177.7 Mb, respectively (Table 1). The unigenes were annotated based on sequence similarities to genes in public databases, including the National Center for Biotechnology Information non-redundant (NCBI-nr) protein database [13], The Arabidopsis Information Resource (TAIR) [14], Kyoto Encyclopedia of Genes and Genomes (KEGG) database [15], and the Gene Ontology (GO) database [16]. We determined that 104,191 (57.90 %), 131,724 (73.20 %), 76,749 (42.60 %), and 67,049 (37.30 %) unigenes matched sequences in the NCBI-nr, TAIR, GO, and KEGG databases, respectively (Table 2).

**Table 2:** Summary of the annotated unigenes

Database	Total unigenes	Annotated unigenes	Percent (%)
NR	179,951	104,191	57.90 %
TAIR	179,951	131,724	73.20 %
GO	179,951	76,749	42.60 %
KEGG	179,951	67,049	37.30 %

### Identification and functional classification of genes differentially expressed among the yellow-green, albescent, and re-greening stages

Three independent biological replicates were analyzed for each stage to evaluate the reproducibility of our results. By comparing the read numbers per unigene in each sample, biological replicates of the nine samples were validated using scatter plots generated with CummeRbund [17]. The plots revealed that the nine RNA-seq samples clustered into three groups of three replicate samples according to their specific stage (Fig. 1b). This result suggests that the three biological replicates generated consistent gene expression results for each stage. Our data also indicated that the unigene expression levels in leaves differed among stages.

We detected the expression of up to 142,798 unigenes in the YG stage, 150,130 unigenes in the W stage, and 140,914 unigenes in the G stage. The unigene expression levels were measured in terms of fragments per kilobase of exon per million mapped reads (FPKM). Putative DEGs were identified considering a false discovery rate (FDR) of less than 0.05. For the pairwise comparisons among the three stages, we detected 15,320 (YG vs G), 9,476 (W vs G), and 8,790 (YG vs W) DEGs (Table 3). The greater abundance of DEGs for the comparison between the YG and G stages than for the other comparisons indicated that the transcriptome profiles of these two stages were much more distinct than those of the other stages (Table 3).

**Table 3:** Number of differentially expressed genes identified by comparing the gene expression levels between two stages

Sample	No. of DEGs	No. of up-regulated unigenes	No. of down-regulated unigenes
W vs YG	8,790	4,123	4,667
G vs W	9,476	6,141	3,335
YG vs G	15,320	6,784	8,536

**Fig. 2:**
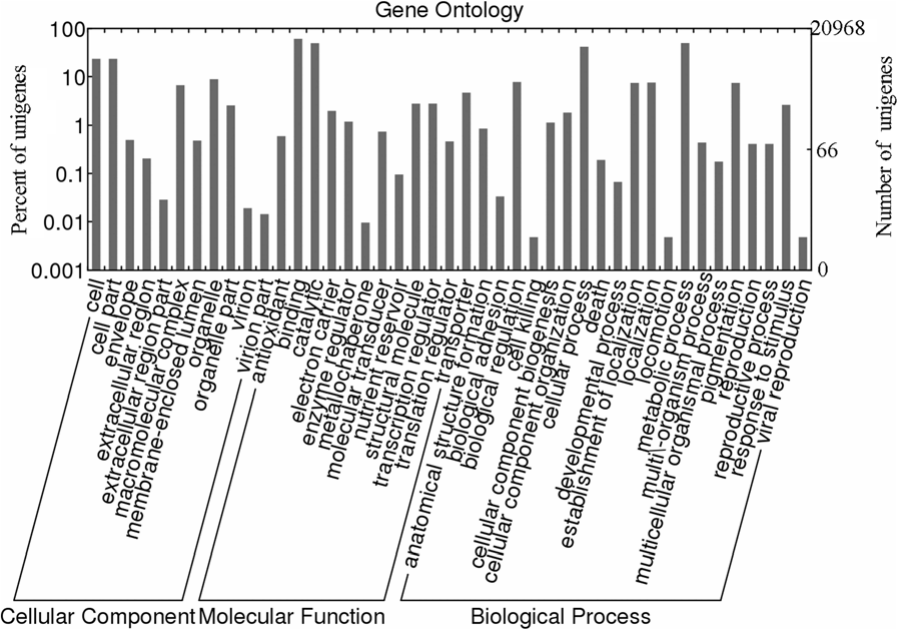
Gene Ontology (GO) classification of unigenes. All unigenes were classified using the biological process, cellular component, and molecular function categories. The y-axis indicates the number and percentage of unigenes assigned to a given GO annotation term

Based on GO analysis, 10,445 (5.8 %) and 1,589 (0.9 %) unigenes were involved in metabolic processes (GO:0008152) and pigmentation (GO:0043473), respectively (Fig. 2). An enrichment analysis of the DEGs revealed the significantly up- or down-regulated GO terms between each pair of stages (Additional files 1 and 2). For the W vs YG comparison, the expression levels of 4,123 unigenes were significantly up-regulated, while the expression levels of 4,667 unigenes were significantly down-regulated (Table 3). The up-regulated DEGs were enriched in GO terms such as glycine dehydrogenase (decarboxylating) activity, oxidoreductase activity, photosystem I, terpenoid biosynthetic process, and photosystem I reaction center. In contrast, the down-regulated DEGs were enriched in the GO terms argininosuccinate synthase activity, transcription coactivator activity, arginine biosynthetic process, purine ribonucleoside monophosphate biosynthetic process, ribonucleoside diphosphate reductase activity, and glutamate synthase activity. For the G vs W comparison, 6,141 and 3,335 unigenes were up- and down-regulated, respectively. The up-regulated DEGs were enriched in GO terms such as terpenoid biosynthetic process, vitamin B6 biosynthetic process, glycine dehydrogenase (decarboxylating) activity, vitamin B6 metabolic process, pyridoxal phosphate biosynthetic process, oxidoreductase activity, and glycolipid biosynthetic process. The down-regulated DEGs were enriched in GO terms including beta-fructofuranosidase activity, sucrose alpha-glucosidase activity, GTP catabolic process, diacylglycerol *O*-acyltransferase activity, acylglycerol *O*-acyltransferase activity, protein polymerization, glycerolipid biosynthetic process, GTP metabolic process, and aminomethyltransferase activity. For the YG vs G comparison, 6,784 and 8,536 unigenes were up- and down-regulated, respectively. The up-regulated DEGs were enriched in GO terms such as oxidoreductase activity, cellular amide metabolic process, glutathione biosynthetic process, GTP cyclohydrolase II activity, terpenoid biosynthetic process, putrescine biosynthetic process, and sulfate adenylyltransferase activity. The down-regulated DEGs were enriched in GO terms including methionine biosynthetic process, outer membrane, DNA replication, oxidoreductase activity, ribonucleoside-diphosphate reductase activity, and GTP catabolic process.

Terpenoids form a large and diverse class of naturally occurring organic chemicals that are extensively used because of their aromatic qualities. In tea plants, terpenoids serve as the main precursors for the aromatic chemicals in tea, and influence pest resistance. The DEGs related to terpenoid biosynthesis were up-regulated from the YG stage to the W stage and then to the G stage. The concentrations of terpenoids, including total carotenoids, β-carotene, and lutein, were lower in the albino tea cultivars than in the normal green tea cultivars [5, 9]. These findings suggest that the terpenoid biosynthetic process was more active in green tea plant leaves.

**Fig. 3:**
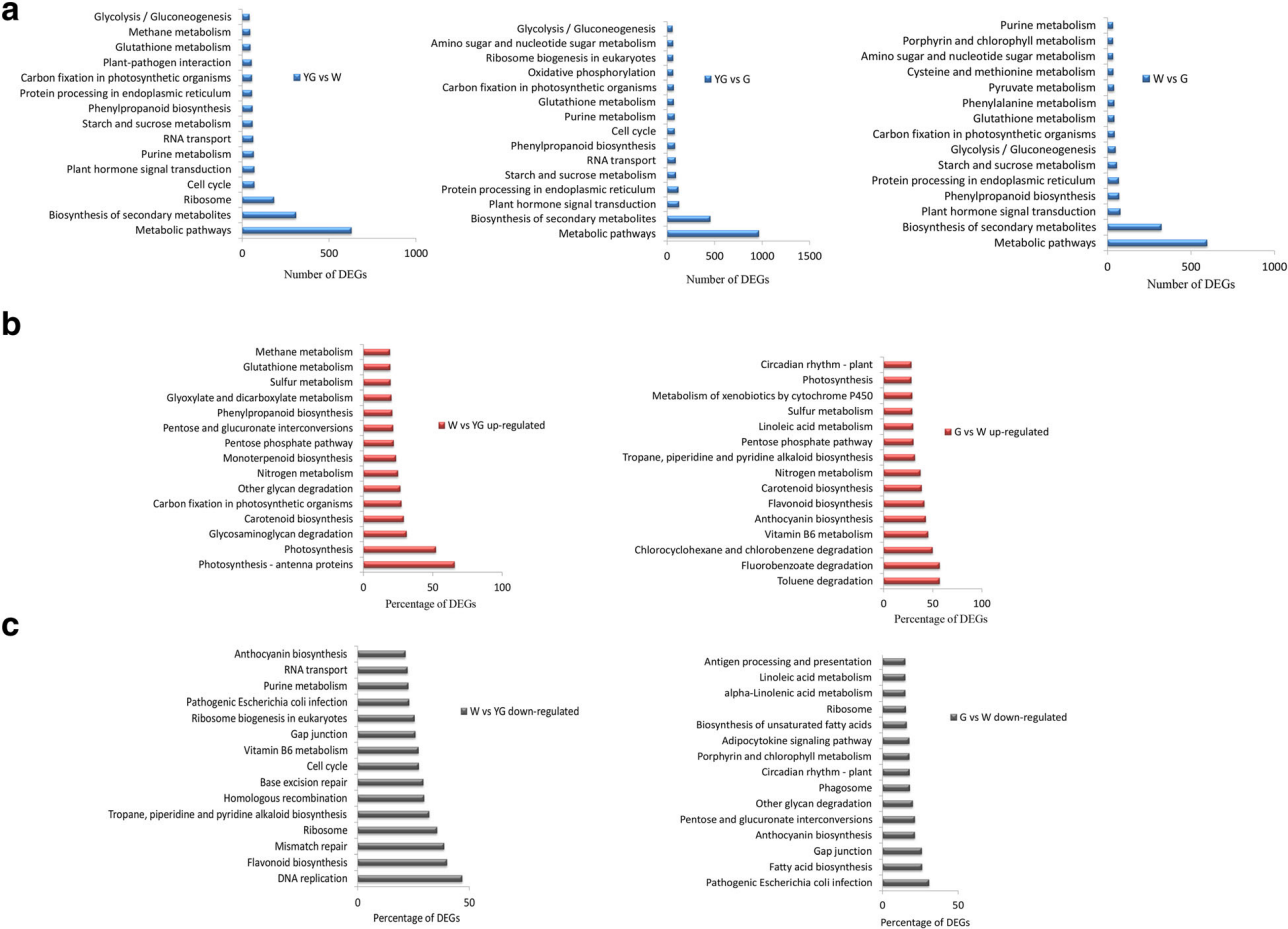
Kyoto Encyclopedia of Genes and Genomes (KEGG) pathway enrichment of differentially expressed unigenes. **a** Top 15 KEGG pathways containing the most differentially expressed unigenes. **b** Top 15 KEGG pathways containing the greatest percentage of up-regulated unigenes from the yellow-green (YG) stage to the albescent (W) stage, and from the W stage to the re-greening (G) stage. **c** Top 15 KEGG pathways containing the greatest percentage of down-regulated unigenes from the YG stage to the W stage, and from the W stage to the G stage. The percentages were calculated relative to the total number of unigenes for a given KEGG pathway

The DEGs between each pair of stages were enriched in genes related to distinct KEGG pathways (Fig. 3). The top 15 KEGG pathways corresponding to the most abundant DEGs are presented in Fig. 3a. With the color and developmental changes in leaves from the YG stage to the W stage and then to the G stage, the KEGG pathways that were enriched in DEGs remained essentially unchanged. The shared KEGG pathways for the YG vs W, W vs G, and YG vs G comparisons included metabolic pathways, biosynthesis of secondary metabolites, starch and sucrose metabolism, phenylpropanoid biosynthesis, purine metabolism, carbon fixation in photosynthetic organisms, and glycolysis or gluconeogenesis. The most abundant up- (Fig. 3b) or down-regulated (Fig. 3c) DEGs between two consecutive stages were assigned to 15 individual KEGG pathways. The common up-regulated KEGG pathways for the W vs YG and G vs W comparisons were related to photosynthesis, carotenoid biosynthesis, nitrogen metabolism, pentose phosphate pathway, and sulfur metabolism. The common down-regulated KEGG pathways for the W vs YG and G vs W comparisons were associated with ribosomes, gap junctions, and anthocyanin biosynthesis.

In photosynthetic organisms, leaves are the site of photosynthesis or carbon fixation, which make them specialized tissues for light-energy absorption, CO_2_ uptake and assimilation, and reduction reactions to form carbohydrates such as glucose. The expression levels of unigenes involved in carbon fixation in photosynthetic organisms exhibited distinct patterns during the leaf color and developmental changes occurring from the YG stage to the W stage and then to the G stage (Fig. 3a). These expression patterns may be responsible for the differences between stages regarding the abundance of metabolites related to carbon fixation [8]. The enhanced expression of DEGs related to photosynthesis may lead to increased carbohydrate synthesis and storage of absorbed energy in the form of starch or sucrose, thereby providing an energy source for tea plant survival and development [18]. The DEGs during the W stage in ‘Anji Baicha’ tea plants were also related to energy metabolism [3, 6, 7].

The secondary metabolites in tea plants contribute to the rich, clean flavors and nutrient contents of tea [19, 20]. These secondary metabolites also have beneficial health effects for humans. Our data indicated that the identified DEGs were related to the biosynthesis of secondary metabolites, which is consistent with the results of a microarray analysis [6]. These observations suggest that the biosynthesis of secondary metabolites is differentially regulated throughout the color and developmental stages in leaves. The DEGs involved in carotenoid biosynthesis were significantly up-regulated from the YG stage to the W stage and then to the G stage (Fig. 3b). The total carotenoid contents of albino cultivars are lower than those of normal green tea cultivars [5, 9]. The DEGs associated with carotenoid biosynthesis might affect the differences in the total carotenoid contents between stages.

Flavonoids are a group of plant polyphenolic secondary metabolites, which includes flavones, flavonols, isoflavones, flavanones, flavanols, and anthocyanidins. The flavan-3-ols, or catechins, are the most prominent flavonoid compounds in tea leaves [21, 22]. These compounds contribute to many features that make tea an important part of the human diet. From the YG stage to the W stage, the DEGs involved in flavonoid biosynthesis were mainly down-regulated (Fig. 3c). In contrast, from the W stage to the G stage, these DEGs were mainly up-regulated (Fig. 3b). While in the pale white shoot of ‘Anji Baicha’, the DEGs involved in flavonoid biosynthesis were down-regulated [6]. This result was contrary to our study which might be because the different tissues used in the two studies. A previous study revealed that the expression of genes encoding the key flavonoid biosynthesis enzymes decreased during the albescent stage, and that the abundance of most catechins was lower in the albescent stage than in the green stage [23]. The dynamic alterations in the expression of the flavonoid biosynthesis-related DEGs may contribute to the differences in catechin abundance among various color and developmental stages.

### Carotenoid biosynthesis

**Fig. 4:**
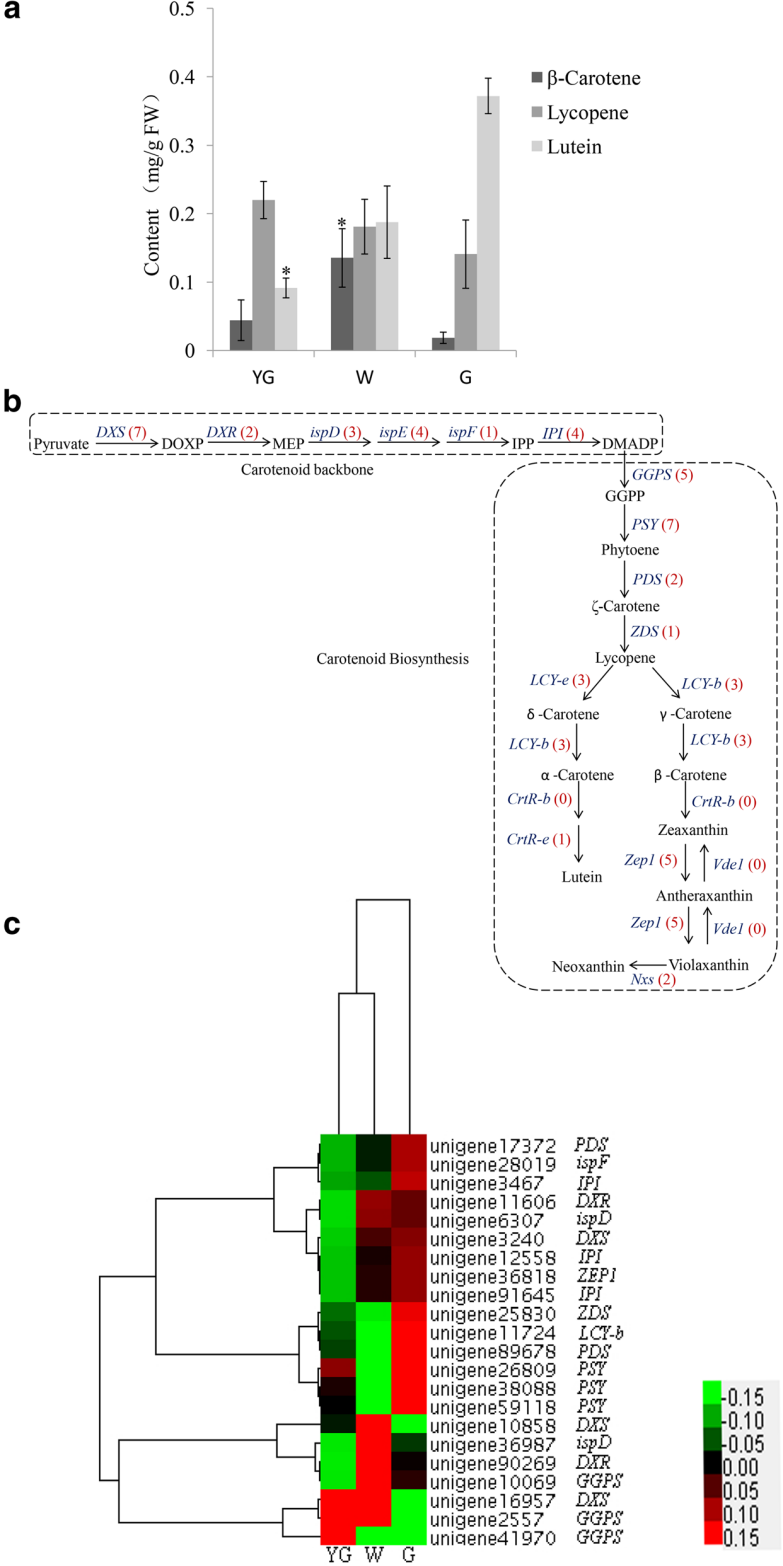
Carotenoid concentrations and carotenoid biosynthesis unigenes at different stages. **a** The concentrations of β-carotene, lycopene, and lutein were determined at different stages. The asterisk indicates a significant difference between the YG or W stages and the G stage (*P* < 0.05; Student’s t-test). **b** Carotenoid biosynthesis pathway. The bracketed numbers in red following each gene name indicate the number of corresponding unigenes identified in our database. **c** All differentially expressed genes involved in carotenoid biosynthesis were hierarchically clustered and mapped using the fragments per kilobase of exon per million mapped reads values. Colors indicate the normalized signal intensity as defined in the bar

Carotenoids play vital roles during photosynthesis [24] and serve as precursors to abscisic acid [25, 26]. In tea plants, carotenoids are present as yellow pigments in fresh leaves and degrade into flavored terpenoids during the production of black tea [27]. Gas chromatography coupled to time-of-flight mass spectrometry (GC-TOF-MS) analyses were conducted to determine the concentrations of β-carotene, lycopene, and lutein at different stages. Compared with the levels during the G stage, significantly higher β-carotene concentrations were observed in the W stage, while a significantly lower lutein concentration was detected in the YG stage (Fig. 4a). The β-carotene, lycopene, and lutein concentration trends differed considerably between the YG and G stages. The β-carotene concentration increased from the YG stage to the W stage, and then decreased from the W stage to the G stage. However, the lycopene and lutein concentrations increased from the YG stage to the G stage (Fig. 4a). Changes in carotenoid abundance are related to altered expression of carotenoid biosynthesis genes [28, 29]. In our database, 51 unigenes were annotated as key genes encoding enzymes related to carotenoid biosynthesis (Fig. 4b and Additional file 3). The expression profiles of all DEGs implicated in carotenoid biosynthesis were hierarchically clustered and plotted in a heat map (Fig. 4c).

Isopentenyl diphosphate (IPP) forms the backbone of carotenoids. In the 1-deoxyxylulose 5-phosphate (DOXP) pathway, IPP is produced from pyruvate and glyceraldehyde-3-phosphate, with DOXP synthase (DXS) functioning as the first enzyme in this pathway. In tomato plants, DXS is involved in a regulatory step during carotenoid biosynthesis [30]. In *Arabidopsis thaliana*, a *DXS* mutation results in the inhibition of chloroplast development, a lack of chlorophyll and carotenoids, and an albino phenotype in seedlings [31]. In our database, seven candidate unigenes encoded DXS, and the expression levels of three of these unigenes (unigene 16957, unigene 10858, and unigene 3240) were significantly different among the various color and developmental stages (Fig. 4c). Two unigenes (unigene 16957 and unigene 10858) were significantly up-regulated in the W stage, while one unigene (unigene 3240) was significantly up-regulated in the G stage. In the next carotenoid biosynthesis step, geranylgeranyl diphosphate synthase (GGPS) catalyzes the addition of three IPP molecules to dimethylallyl diphosphate. Our database contained five unigenes that were homologous to *GGPS*. Two of these unigenes (unigene 41970 and unigene 2557) were significantly up-regulated during the YG stage, while one (unigene 10069) was significantly up-regulated in the W stage.

In the carotenoid biosynthesis pathway, phytoene synthase (PSY) catalyzes the synthesis of phytoene from two GGPP molecules [32] (Fig. 4b). Phytoene synthase is a rate-limiting enzyme of carotenoid biosynthesis in marigold flowers [33], canola (*Brassica napus*) seeds [34], and ripening tomato fruits [28, 35]. Of the seven candidate *PSY* unigenes identified in our study, the expression levels of three (unigene 59118, unigene 26809, and unigene 38088) were significantly up-regulated in the G stage (Fig. 4c). Lycopene β-cyclase (LCY-b) activity results in the addition of a β-ionone ring at each end of the lycopene molecule to generate β-carotene. Additionally, lycopene ε-cyclase generates an ε-ring to produce δ-carotene. We detected four unigenes homologous to *LCY*-*b* in the tea plant transcriptome, only one of which (unigene 11724) was up-regulated in the G stage (Fig. 4c). In tomato plants, *LCY*-*b* is actively expressed in green tissues [36, 37]. Although *LCY*-*b* was highly expressed during the G stage, β-carotene concentrations were lowest in this stage (Fig. 4a). This inconsistency between the gene expression level and metabolite concentration might be due to post-transcriptional regulation of *LCY*-*b*, leading to inhibited enzymatic activity.

Zeaxanthin epoxidase (Zep1) catalyzes the conversion of zeaxanthin to violaxanthin via antheraxanthin [38]. We identified five unigene candidates for *Zep1*, only one of which (unigene 36818) was expressed more during the G stage than in the other two stages. Violaxanthin deepoxidase (VDE) converts violaxanthin back to zeaxanthin [39]. We did not identify any candidate unigenes for *VDE*, which might not be expressed in the three tea leaf color stages or might be expressed at levels below the limit of detection. There are two types of neoxanthin synthase (NXS) in plants. One is nearly identical to lycopene β-cyclase, which can convert lycopene into β-carotene and violaxanthin into neoxanthin. The other form can only convert violaxanthin to neoxanthin [40]. In our database, the sequences of two unigenes (unigene 40018 and unigene 14302) were highly similar to that of *NXS*, but neither unigene exhibited significantly different expression levels among the three analyzed stages.

We calculated the Spearman’s correlation coefficients between the expression levels of DEGs and concentrations of β-carotene, lycopene, and lutein at each stage. The expression levels of many unigenes were significantly correlated with metabolite concentrations (Table 4). We identified one *PSY*-encoding unigene (unigene 26809) whose expression level was significantly correlated with β-carotene concentrations. Additionally, the expression levels of 12 unigenes were significantly correlated with lycopene concentrations. These unigenes encoded DXS, DXR, ispF, IPI, GGPS, PSY, LCY-B, PDS, ZDS, and ZEP. We also detected 12 unigenes whose expression levels were significantly correlated with lutein concentrations, including unigenes that were annotated as *DXS*, *DXR*, *ispD*, *ispF*, *IPI*, *GGPS*, *PSY*, *LCY*-*B*, *PDS*, *ZDS*, and *ZEP*. These unigenes might be responsible for the differences in carotenoid biosynthesis during the various ‘Anji Baicha’ leaf color and developmental stages.

**Table 4:** Correlation coefficients between expression levels of differentially expressed carotenoid biosynthetic pathway unigenes and concentrations of β-carotene, lycopene, and lutein

Unigene	Gene name	β-Carotene	Lycopene	Lutein
unigene3240	*DXS*	0.865	0.016^*^	0.02^*^
unigene10858	*DXS*	0.332	0.406	0.865
unigene16957	*DXS*	0.224	0.516	0.077
unigene11606	*DXR*	0.332	0.002^**^	0.01^**^
unigene90269	*DXR*	0.256	0.587	0.678
unigene36987	*ispD*	0.865	0.244	0.05^*^
unigene6307	*ispD*	0.346	0.769	0.732
unigene28019	*ispF*	0.265	0.002^**^	0.002^**^
unigene3467	*IPI*	0.332	0.004^**^	0.001^**^
unigene12558	*IPI*	0.516	0.013^*^	0.02^*^
unigene91645	*IPI*	0.557	0.967	0.253
unigene10069	*GGPS*	0.865	0.036^*^	0.332
unigene2557	*GGPS*	0.576	0.898	0.46
unigene41970	*GGPS*	1	0.205	0.036^*^
unigene26809	*PSY*	0.05^*^	0.02^*^	0.17
unigene59118	*PSY*	0.576	0.476	0.178
unigene38088	*PSY*	0.244	0^**^	0.002^**^
unigene11724	*LCY*-*B*	0.332	0^**^	0.002^**^
unigene17372	*PDS*	0.332	0.001^**^	0.04^*^
unigene89678	*PDS*	0.637	0.421	0.465
unigene25830	*ZDS*	0.308	0^**^	0.005^**^
unigene36818	*ZEP*	0.332	0^**^	0.002^**^

*Significant difference at *P* ≤ 0.05; **Significant difference at *P* ≤ 0.01

### Chlorophyll biosynthesis

**Fig. 5:**
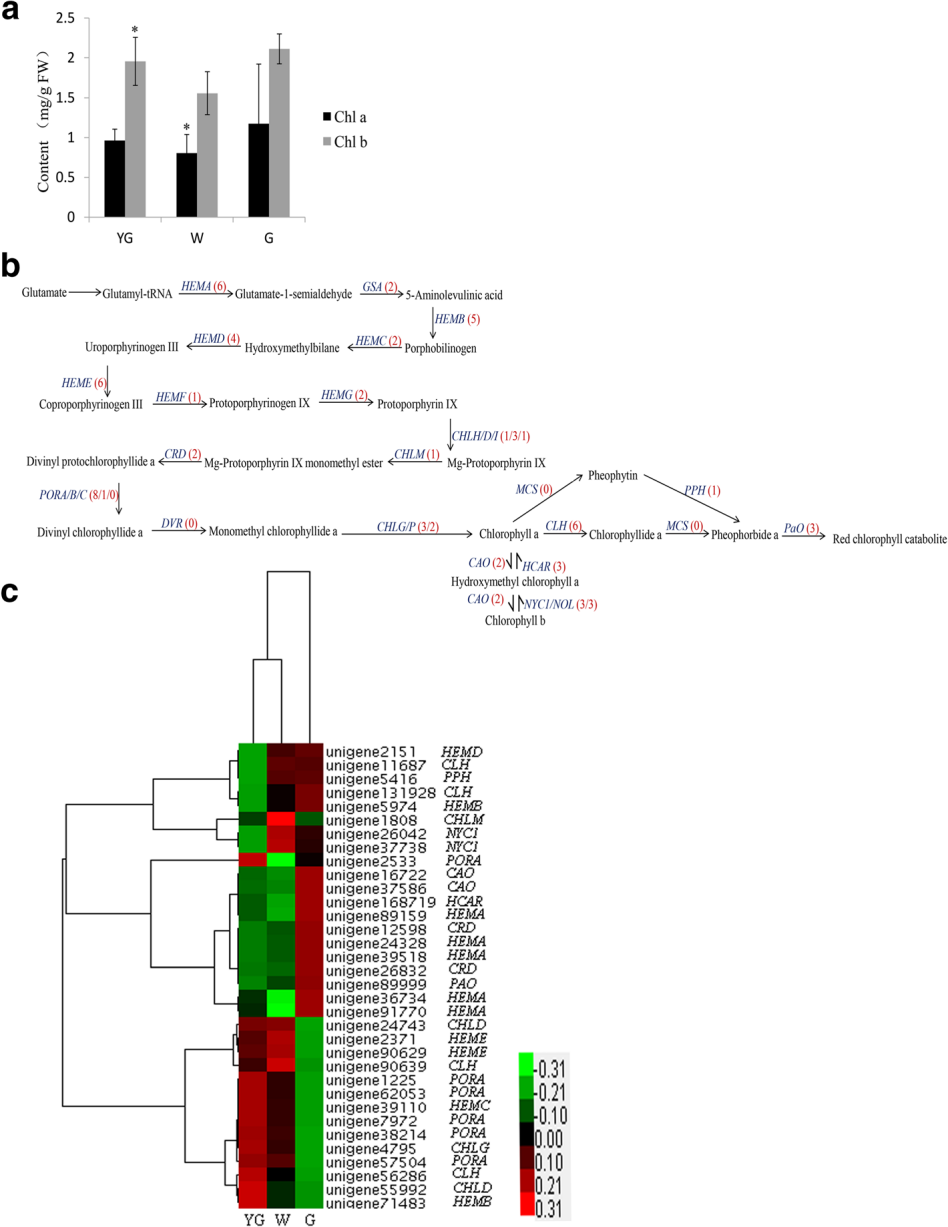
Chlorophyll concentrations and the related unigenes. **a** The chlorophyll *a* and *b* concentrations were determined at different stages. The asterisk indicates a significant difference between the YG or W stages and the G stage (*P* < 0.05; Student’s t-test). **b** Chlorophyll biosynthesis pathway. The bracketed numbers in red following each gene name indicate the number of corresponding unigenes identified in our database. **c** All differentially expressed genes involved in chlorophyll biosynthesis were hierarchically clustered and mapped using the fragments per kilobase of exon per million mapped reads values. Colors indicate the normalized signal intensity as defined in the bar

Chlorophylls are essential for photosynthesis, light harvesting, and energy transduction. Additionally, chlorophylls are Mg^2+^-containing tetrapyrrole pigments responsible for turning plants green. The albino phenotype in tea plants is a result of a lack of chlorophylls during shoot development. In the early spring, the development of chloroplasts from etioplasts and the accumulation of chlorophylls *a* and *b* are blocked in the new shoots of ‘Anji Baicha’ tea plants [2]. The chlorophyll *a* and *b* concentrations were determined at each stage. Compared with those of the G stage, the chlorophyll concentrations in the W and YG stages were significantly lower (Fig. 5a). Additionally, the chlorophyll *a* and *b* concentrations decreased from the YG stage to the W stage, and then increased from the W stage to the G stage (Fig. 5a).

**Fig. 6:**
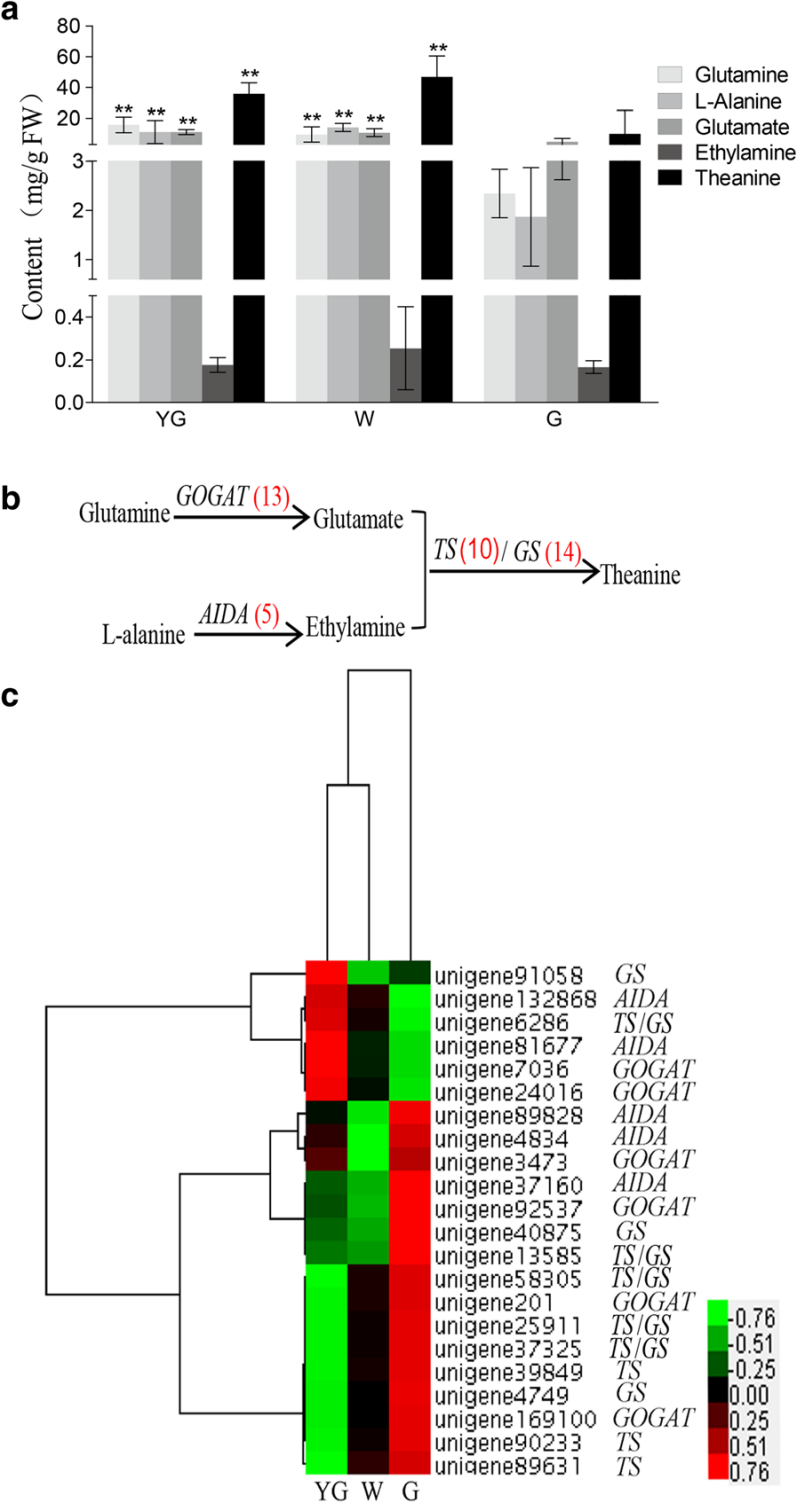
Theanine biosynthesis pathway and the related unigenes. **a** The glutamine, glutamate, alanine, ethylamine, and theanine concentrations were determined at different stages. The double asterisks indicate a significant difference between the YG or W stages and the G stage (*P* < 0.01; Student’s t-test). **b** Theanine biosynthesis pathway. The bracketed numbers in red following each gene name indicate the number of corresponding unigenes identified in our database. **c** All differentially expressed genes involved in theanine biosynthesis were hierarchically clustered and mapped using the fragments per kilobase of exon per million mapped read values. Colors indicate the normalized signal intensity as defined in the bar

The chlorophyll metabolic pathway includes the following three phases: biosynthesis of chlorophyll *a*, interconversion between chlorophylls *a* and *b*, and degradation of chlorophyll *a* [41–44]. In our database, 71 unigenes were related to chlorophyll biosynthesis, including the genes for almost all of the key enzymes (Fig. 5b and Additional file 4). The expression levels of the DEGs were determined via a hierarchical cluster analysis (Fig. 5c). The expression levels of the gene encoding geranylgeranyl diphosphate reductase (*CAO*), which is an enzyme involved in chlorophyll *b* biosynthesis, are elevated in plants with insufficient amounts of chlorophyll *b* [45]. Two *CAO* unigenes (unigene 16722 and unigene 37586) were identified in our database, and both were significantly up-regulated in the G stage. This finding implies that high *CAO* expression levels might induce the efficient biosynthesis of chlorophyll *b* to increase its concentration during the G stage (Fig. 5a). *NYC1* encodes chlorophyll *b* reductase, which catalyzes the degradation of chlorophyll *b* to 7-hydroxymethyl chlorophyll *a* [46, 47]. The degradation of chlorophyll *b* is suppressed in *NYC1* mutant plants, which remain green until just before death due to natural senescence [48]. Three *NYC1* unigenes were detected in our database, and the expression levels of two of these (unigene 37738 and unigene 26042) were significantly higher in the W stage than in the YG and G stages. High *NYC1* unigene expression levels during the W stage might enhance the degradation of chlorophyll *b* (Fig. 4c). In plants, the NOL (NYC1-like) protein is closely related to NYC1, and *NOL* mutant plants also remain green, similar to wild-type plants [49]. Three *NOL* unigenes were identified in our database, but the expression levels of these unigenes were not significantly different between the YG and G stages. Hydroxymethyl chlorophyll *a* reductase (HCAR) converts 7-hydroxymethyl chlorophyll *a* to chlorophyll *a* [50]. The *HCAR* expression levels are strongly correlated with chlorophyll content in carnation flower petals [51]. Additionally, *HCAR* expression is strongly up-regulated during the stage in which etiolated *A. thaliana* seedlings turn green [52]. These results suggest that HCAR is essential for chlorophyll turnover during the greening stage. Three *HCAR* unigenes were identified in our database, and only one (unigene 168719) was significantly up-regulated during the G stage. High expression levels of this unigene in the G stage might contribute to the higher chlorophyll *a* concentrations during this stage compared with the other two stages (Fig. 5a). Pheophytinase (PPH) has a key function in chlorophyll degradation. The expression of *PPH* is induced in darkness, which accelerates chlorophyll degradation. In *PPH* mutant plants, chlorophyll degradation is inhibited, and the plants exhibit a sustained green phenotype during senescence [53]. In our study, the expression of *PPH* (unigene 5416) was lower in the YG stage than in the W and G stages (Fig. 5b), suggesting the lowest chlorophyll *a* degradation rate occurred during the YG stage. However, the chlorophyll *a* concentration in the YG stage was lower than that during the G stage. This may have been because the expression level for unigene 4795, which encodes chlorophyll synthase (CHLG), was significantly higher in the G stage than in the YG stage (Fig. 5c). The higher chlorophyll *a* concentration in the G stage than in the YG stage might be because chlorophyll *a* was synthesized faster than it was degraded. Chlorophyllase (CLH) catalyzes the conversion of chlorophyll *a* to chlorophyllide *a*. In citrus plants, *CLH* expression levels are negatively correlated with chlorophyll contents [54]. We identified six candidate *CLH* unigenes in our database (Fig. 5b), four of which were significantly differentially expressed between the YG and G stages. The expression levels of unigene 56286 and unigene 90639 were up-regulated during the YG and W stages, respectively, while two unigenes were up-regulated in the G stage (unigene 11687 and unigene 131928) (Fig. 6c). Unigene 56286 and unigene 90639 might contribute to chlorophyll *a* degradation to lower the chlorophyll concentration more during the YG and W stages than in the G stage. The expression levels of *PaO*, which encodes pheophorbide a oxygenase, are closely correlated with the rate of chlorophyll breakdown [55]. Three *PaO* unigenes were detected in our database, but only one (unigene 89999) was significantly up-regulated from the YG stage to the G stage. However, the chlorophyll content in the G stage was relatively high (Fig. 5a). Identical results were reported for carnations [51], potentially because the post-transcriptional regulation of *PaO* inhibits the activity of the encoded enzyme [56] or because chlorophyll *a* is synthesized faster than it is degraded.

Spearman’s correlation coefficients between the expression levels of DEGs and concentrations of chlorophylls *a* and *b* were calculated for each stage. The expression levels of two and three unigenes were significantly correlated with chlorophyll *a* and *b* concentrations, respectively (Table 5). The *PORA* (unigene 2533) expression levels were significantly correlated with chlorophyll *a* concentrations. The expression of two unigenes (unigene 26042 and unigene 37738) encoding *NYC1* was significantly correlated with the concentration of chlorophylls *a* and *b*. These unigenes might be responsible for the differences in the biosynthesis of chlorophylls *a* and *b* among the different leaf color and developmental stages of ‘Anji Baicha’ tea plants.

**Table 5:** Correlation coefficients between expression levels of differentially expressed chlorophyll biosynthetic pathway unigenes and concentrations of chlorophylls *a* and *b*

Unigene	Gene Name	Chl a	Chl b
unigene39518	*HEMA*	0.102	0.364
unigene36734	*HEMA*	0.634	0.282
unigene24328	*HEMA*	0.498	0.25
unigene89159	*HEMA*	0.031	0.188
unigene91770	*HEMA*	0.429	0.763
unigene5974	*HEMB*	0.123	0.123
unigene71483	*HEMB*	0.282	0.24
unigene39110	*HEMC*	0.282	0.513
unigene2151	*HEMD*	0.23	0.158
unigene2371	*HEME*	0.618	0.813
unigene90629	*HEME*	0.511	0.024
unigene24743	*CHLD*	0.634	0.897
unigene55992	*CHLD*	0.24	0.166
unigene1808	*CHLM*	0.22	0.108
unigene26832	*CRD*	0.339	0.364
unigene12598	*CRD*	0.402	0.282
unigene2533	*PORA*	0.044^*^	0.339
unigene38214	*PORA*	0.201	0.603
unigene1225	*PORA*	0.282	0.513
unigene62053	*PORA*	0.201	0.603
unigene7972	*PORA*	0.796	0.376
unigene57504	*PORA*	0.183	0.166
unigene4795	*CHLG*	0.121	0.282
unigene16722	*CAO*	0.327	0.22
unigene37586	*CAO*	0.23	0.364
unigene131928	*CLH*	0.308	0.204
unigene11687	*CLH*	0.074	0.351
unigene90639	*CLH*	0.164	0.482
unigene56286	*CLH*	0.25	0.813
unigene168719	*HCAR*	0.587	0.415
unigene26042	*NYC1*	0.011^*^	0.015^*^
unigene37738	*NYC1*	0.007^**^	0.01^**^
unigene5416	*PPH*	0.012	0.249
unigene89999	*PAO*	0.123	0.123

*Significant difference at *P* ≤ 0.05; **Significant difference at *P* ≤ 0.01

### Theanine biosynthesis

Theanine synthetase (TS) catalyzes the synthesis of theanine from glutamic acid and ethylamine [57, 58] (Fig. 6b). Additional enzymes involved in theanine biosynthesis include glutamate synthase (GOGAT) and alanine decarboxylase (AIDA), which catalyze the conversion of glutamine to glutamate and alanine to ethylamine, respectively. Glutamine synthetase (GS) is highly homologous to TS, and can also catalyze the conversion of glutamic acid and ethylamine to theanine [59]. Ultra-performance liquid chromatography–triple quadrupole mass spectrometry (UPLC–QqQ-MS) analyses were conducted to determine the concentrations of glutamine, glutamate, alanine, and theanine. Additionally, GC-TOF-MS was used to measure the ethylamine concentration at different stages. Except for ethylamine, all metabolites were detected at significantly higher concentrations in the YG and W stages than in the G stage (Fig. 6a). The glutamine and glutamate concentrations decreased from the YG stage to the G stage. In contrast, the alanine and theanine concentrations increased from the YG stage to the W stage, and then decreased from the W stage to the G stage (Fig. 6a).

All unigenes involved in theanine biosynthesis were identified (Fig. 6b and Additional file 5). The DEGs related to theanine biosynthesis were hierarchically clustered and plotted in a heat map (Fig. 6c). The G stage consisted of many highly expressed DEGs, including all unigenes encoding the key enzymes in the theanine biosynthesis pathway. These results suggest that the G stage is an important phase for theanine biosynthesis. One substrate in the theanine biosynthesis pathway is ethylamine, which is produced via the decarboxylation of alanine by AIDA [60]. Because the *AIDA* gene are specific to tea plant and has not been identified and functionally characterized, the *AIDA* genes used in our study encoded arginine decarboxylases, and contained domains similar to those in *AIDA* [61, 62]. A total of five putative *AIDA* unigenes were identified. Among them, two (unigene 132868 and unigene 81677) were significantly up-regulated in the YG stage, while the remaining three (unigene 89828, unigene 4834, and unigene 37160) were significantly up-regulated in the G stage. Another substrate in the theanine biosynthesis pathway is glutamate, which is produced from glutamine by GOGAT. In our database, 13 putative *GOGAT* unigenes were identified. Two of these unigenes (unigene 7036 and unigene 24016) were significantly up-regulated in the YG stage, and four unigenes (unigene 3473, unigene 92537, unigene 201, and unigene 169100) were significantly up-regulated during the G stage. These results indicated that there are differences in the expression patterns of unigenes from the same family. Additionally, high unigene expression levels do not necessarily correspond to high metabolite concentrations. Increased glutamate levels lead to up-regulated theanine biosynthesis in tea seedlings [63]. In our study, both glutamate and theanine were significantly more abundant in the W and YG stages than in the G stage. A previous study reported that glutamate and theanine concentrations are higher in albino cultivars than in normal green cultivars [9]. Glutamate also participates in chlorophyll synthesis (Fig. 5a) [42]. The suppression of chlorophyll biosynthesis during the W stage might lead to elevated levels of glutamate, thereby increasing theanine biosynthesis. Theanine synthetase is unique to tea plants, and 10 candidate *TS* unigenes were identified in our database. Eight *TS* unigenes (unigene 39849, unigene 89631, unigene 40875, unigene 13585, unigene 58305, unigene 25911, unigene 37325, and unigene 90233) were up-regulated, and one (unigene 6286) was down-regulated in the G stage. In tea plants, *TS* genes are highly homologous to *GS* genes [57]. Fourteen *GS* unigenes were identified in our database, six of which were also annotated as *TS* genes. Two of the *GS* unigenes were significantly up-regulated in the YG stage, while six were significantly up-regulated in the G stage (Fig. 6c).

We calculated the Spearman’s correlation coefficients between expression levels of DEGs and concentrations of ethylamine, glutamine, and theanine during each stage (Table 6). The expression level of *GOGAT*, which is encoded by unigene 201, was significantly correlated with ethylamine concentrations. The expression levels of five unigenes (corresponding to *GOGAT*, *AIDA*, *GS*, and *TS*) were significantly correlated with glutamine concentrations. *AIDA* expression levels (unigene 37160) were correlated with theanine concentrations. These unigenes might be responsible for the variability in the biosynthesis of the associated metabolites in the different ‘Anji Baicha’ leaf color and developmental stages.

**Table 6:** Correlation coefficients between expression levels of differentially expressed theanine biosynthetic pathway unigenes and concentrations of ethylamine, glutamine, and theanine

Unigene	Gene Name	Ethylamine	Glutamate	Theanine
unigene24016	*GOGAT*	0.088	0.077	0.205
unigene201	*GOGAT*	0.042^*^	0.013^*^	0.205
unigene3473	*GOGAT*	0.7	0.637	0.406
unigene169100	*GOGAT*	0.088	0.025^*^	0.205
unigene7036	*GOGAT*	0.168	0.402	0.815
unigene92537	*GOGAT*	0.725	0.725	0.272
unigene132868	*AIDA*	0.798	0.016^*^	0.139
unigene37160	*AIDA*	0.516	0.099	0.02^*^
unigene81677	*AIDA*	0.224	0.966	0.265
unigene89828	*AIDA*	0.367	0.515	0.681
unigene4834	*AIDA*	0.061	0.94	0.556
unigene13585	*GS*	0.637	0.308	0.286
unigene25911	*GS*	0.058	0.077	0.244
unigene37325	*GS*	0.125	0.025^*^	0.154
unigene40875	*GS*	0.898	0.898	0.831
unigene91058	*GS*	0.865	0.488	0.637
unigene6286	*GS*	0.604	0.539	0.025
unigene58305	*GS*	0.515	0.815	0.025
unigene4749	*GS*	0.092	0.861	0.907
unigene39849	*TS*	0.077	0.03^*^	0.17
unigene90233	*TS*	0.515	0.681	0.147
unigene89631	*TS*	0.127	0.272	0.476

*Significant difference at *P* ≤ 0.05

### Validation of the expression levels of differentially expressed genes at different stages

**Fig. 7:**
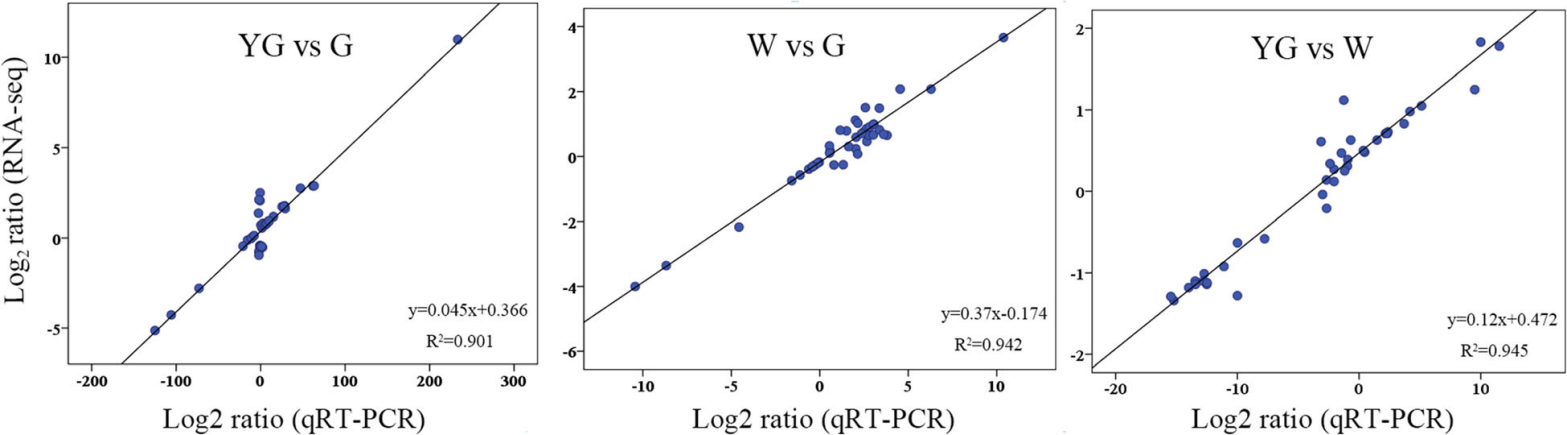
Verification of RNA-sequencing results using quantitative reverse transcription polymerase chain reaction (qRT-PCR) assays. Forty-two differentially expressed genes were selected from the carotenoid, chlorophyll, and theanine biosynthesis pathways. Scatter diagrams show the correlations of the log ratios (log_2_ fold-change) of the unigene expression levels as measured by qRT-PCR and RNA-sequencing. The qRT-PCR data were normalized using the ‘housekeeping’ gene *GAPDH*. The qRT-PCR primers are listed in Additional file 6

To validate the expression-level changes in DEGs as reflected by FPKM values in different stages, we selected 42 unigenes (11 carotenoid biosynthesis unigenes, 22 chlorophyll metabolism unigenes, and nine theanine biosynthesis unigenes) and analyzed their expression levels in the YG, W, and G stages using quantitative reverse transcription polymerase chain reaction (qRT-PCR). Pearson’s correlation coefficients were used to determine the correlations of the gene expression level fold-changes between each pair of examined stages as measured by RNA-seq and qRT-PCR. The results revealed strong correlations (*R*^2^ > 0.9) between the RNA-seq and qRT-PCR data (Fig. 7). These observations indicated that the gene expression changes as detected by RNA-seq reflect the transcriptome profile differences between stages.

## Conclusions

We completed a transcriptome-level analysis of ‘Anji Baicha’ tea plants at different color and developmental stages. The transcriptome profiles differed considerably among the YG, W, and G stages. The differentially expressed unigenes were mainly related to metabolic pathways, biosynthesis of secondary metabolites, phenylpropanoid biosynthesis, and carbon fixation in photosynthetic organisms. Chemical analyses revealed the theanine and β-carotene levels were higher and the chlorophyll *a* levels were lower in the W stage than in the G stage. Furthermore, we identified unigenes associated with carotenoid, chlorophyll, and theanine biosyntheses, and the expression patterns of the differentially expressed unigenes implicated in these pathways were characterized. Analyses of gene expression levels and metabolite contents enabled the identification of key unigenes whose expression levels may significantly affect metabolite concentrations. These results revealed the relationship between gene expression and the biosynthesis of carotenoids, chlorophyll, and theanine. Our findings have helped to elucidate the molecular mechanisms underlying the biosynthesis of characteristic biochemicals in albino tea plants. The transcriptome-level data generated in this study may be a valuable resource for further molecular and genomic studies of albino tea plants.

## Methods

### Plant materials

Tea plants [*Camellia sinensis* (L.) O. Kuntze cv. ‘Anji Baicha’] were grown in the China National Germplasm Hangzhou Tea Repository of the Tea Research Institute, Chinese Academy of Agricultural Sciences. Leaves of 4-year-old tea plants were collected at 9 to 11 am. In early spring, the new shoots were yellow-green. The YG stage leaves were the first leaves, and were collected on April 1, 2014. When the shoots produced three leaves and one bud or four leaves and one bud, the second or third leaves were white. These W stage leaves were harvested on April 15, 2014. The higher temperatures of the late spring period resulted in green leaves. The G stage leaves (i.e., fifth or sixth leaves) were collected on May 9, 2014. Each sample was individually collected from three tea plants to obtain three biological replicates for RNA-seq or from five tea plants to obtain five biological replicates for chemical component analyses. All collected samples were immediately frozen in liquid nitrogen and stored at −70 °C.

### Library preparation and sequencing

Total RNA was extracted from tea leaves using the RNeasy Plus Mini Kit (Qiagen, Valencia, CA, USA) and then treated with TURBO DNase (Ambion, Austin, TX, USA). The integrity of the isolated RNA was confirmed using the RNA 6000 Nano LabChip kit and a 2100 Bioanalyzer (Agilent Technologies, Palo Alto, CA, USA). The libraries used for sequencing were prepared using a kit from Illumina. First, mRNA was purified from 20 μg total RNA using oligo(dT) magnetic beads. Second, the purified mRNA was cut into short fragments in fragmentation buffer. The short fragments were used as templates to synthesize first-strand cDNA, followed by the synthesis of second-strand cDNA. Third, the QIAquick PCR Extraction Kit (Qiagen) was used to purify the cDNA fragments, which underwent an end-repair process before being ligated to sequencing adaptors. Finally, the resulting products were purified by agarose gel electrophoresis, and the final cDNA libraries were generated following PCR enrichment. The cDNA libraries were sequenced using the Illumina HiSeq™ 2000 sequencing system (Illumina, San Diego, CA, USA).

### Unigene assembly, annotation, and expression analysis

To obtain high-quality reads, raw data were filtered to remove adaptor sequences and reads with unknown or low-quality bases. *De novo* assembly was performed using the Trinity program (release 20130225 [64]). We first combined the reads with default parameters to form fragments longer than 200 bp, which were defined as contigs. The reads were then mapped to the contigs to obtain longer sequences using the paired-end reads as templates. Finally, cap3/PriceTI was used to connect the contigs and obtain sequences that could not be extended on either end. Such sequences were defined as unigenes.

Unigenes were annotated using the BLASTx function of selected protein databases, including the NCBI-nr [13] and TAIR databases [14], considering an E-value threshold of 10^−5^. Pathway analyses were conducted using KAAS [15], while GO classifications were completed using WEGO [65] based on GO annotation terms provided by the Blast2GO program [66]. Gene expression levels were calculated based on FPKM values using Cufflinks (version 1.0.3) [67]. The significance of the gene expression level differences between two stages was assessed via the Student’s t-test using FPKM values obtained from three cDNA libraries for each stage. To identify the DEGs between two stages, a threshold FDR of < 0.05 was used to judge the significance of gene expression differences. Hierarchical cluster analyses were conducted with the normalized FPKM values using complete linkage groupings with the aid of Cluster 3.

### Unigene expression level analysis

Total RNA extracted using the RNeasy Plus Mini Kit was treated with TURBO DNase to remove genomic DNA. First-strand cDNA was synthesized from 1 μg DNA-free RNA in a reverse-transcription reaction using random hexamer primers and the MultiScribe reverse transcriptase from a High Capacity cDNA Reverse Transcription Kit (Applied Biosystems, Foster City, CA, USA). The cDNA samples were diluted 10-fold in nuclease-free water and used as templates for qRT-PCR analysis. The qRT-PCR primer pairs are listed in Additional file 6. All of the analyzed unigenes were examined using three technical replicates and three biological replicates for each stage. The qRT-PCR was conducted using an ABI 7500 Real-Time PCR System (Applied Biosystems), SYBR Green, and a PrimeScript™ RT Reagent qPCR Kit (Takara, Dalian, China). Relative transcript abundances were calculated according to the comparative cycle threshold method, with *GAPDH* as an internal standard [68]. Pearson correlation analyses were completed using the SPSS Statistics 17.0 software package (SPSS Inc., Chicago, IL, USA). Correlation analyses were conducted using the log ratios (i.e., log_2_ fold-change between stages) of unigene expression levels *determined by qRT-PCR and RNA-seq*.

### Chlorophyll content measurements

The chlorophyll contents were measured in five biological replicates according to the method of Arnon [69]. Chlorophylls were extracted from 100-mg leaf samples using 80 % acetone. The extracts were spectrophotometrically analyzed at 645 and 663 nm. The Student’s t-test was used to compare the chlorophyll contents in the YG and W stages with those in the G stage.

### Quantification of leaf metabolites by UPLC–QqQ-MS

For each stage, 100-mg leaf samples were submerged in 500 μL methanol:water (3:1, v/v) with ultrasonic mixing for 5 min. The mixtures were centrifuged at 12,000 rpm for 10 min at 4 °C, and the supernatants were analyzed using a UPLC–QqQ-MS instrument. Five replicates were analyzed for each sample.

A 2-μL aliquot of the supernatant was injected into a Zorbax Eclipse Plus C18 column (50 × 2.1 mm, 1.8-μm particle size) (Agilent Technologies, Palo Alto, CA, USA) that was maintained at 20 °C in an Agilent 6400 Triple Quadrupole LC/MS system (Agilent Technologies, Santa Clara, CA, USA). The mobile phase of the binary gradient elution mixture consisted of (A) aqueous formic acid (0.1 %, v/v) and (B) acetonitrile, and the sample separation was completed using the following gradient: 98 % A and 2 % B from 0 to 2 min, 100 % B from 3 to 7 min, and 98 % A and 2 % B from 8 to 10 min. The flow rate was 0.2 mL/min. All samples were maintained at 4 °C during analysis in the multiple reaction monitoring mode to maximize sensitivity. The source temperature was set at 100 °C, while the desolvation gas temperature was 300 °C. The gas flow rate was 3.0 L/min. Data were analyzed using the Agilent MassHunter Qualitative Analysis B.04.00 software (Agilent Technologies, Santa Clara, CA, USA). Standards for glutamine, glutamate, alanine, and theanine were dissolved in methanol:water (3:1, v/v) for a final concentration of 100 μg/mL. The diluted standards were mixed to generate calibration curves. The glutamine, glutamate, alanine, and theanine concentrations were determined based on the calibration curves. The Student’s t-test was used to compare the metabolite concentrations in the YG and W stages with those in the G stage.

### Quantification of leaf metabolites by GC-TOF-MS

Each 100-mg leaf sample was submerged in 500 μL methanol:water (3:1, v/v) and homogenized in a ball mill at 65 Hz for 3 min. The mixture was then centrifuged at 12,000 rpm for 10 min at 4 °C. A 350-μL aliquot of the supernatant was transferred to a glass sampling vial and vacuum-dried at room temperature for 1.5 h. The dried residue was treated with 80 μL methoxyamine (20 mg/mL in pyridine) and incubated at 80 °C for 20 min, followed by the addition of 80 μL BSTFA (containing 1 % TCMS, v/v) and an incubation at 70 °C for 1 h.

The GC-TOF-MS analysis was conducted using an Agilent 7890 gas chromatography system coupled with a Pegasus HT time-of-flight mass spectrometer. The system included a DB-5MS capillary column coated with 5 % diphenyl cross-linked with 95 % dimethylpolysiloxane (30 m × 250 μm inner diameter, 0.25 μm film thickness; J&W Scientific, Folsom, CA, USA). A 1-μL aliquot of analyte was injected in the splitless mode. Helium was used as the carrier gas. The front inlet purge flow was 3 mL/min, while the gas flow rate through the column was 20 mL/min. The temperature was initially set at 50 °C for 1 min, and then raised to 320 °C at a rate of 10 °C/min. The temperature was held at 320 °C for 5 min. The injection and transfer line temperatures were 280 °C, while the ion source temperature was 220 °C. The energy level was set at −70 eV in the electron impact mode. The mass spectrometry data within the *m*/*z* range of 85–600 were acquired in full-scan mode at a rate of 20 spectra/s after a solvent delay of 366 s.

The β-carotene, lycopene, lutein, and ethylamine standards were dissolved in methanol:water (3:1, v/v) for a final concentration of 100 μg/mL. The diluted standards were mixed and then derivatized and analyzed as described for the leaf samples to produce calibration curves. The β-carotene, lycopene, lutein, and ethylamine concentrations were determined based on the calibration curves, and the concentrations in the YG and W stages were compared with those in the G stage using the Student’s t-test.

### Correlation analysis

Correlation analyses were completed according to Spearman’s parametric correlation test in the SPSS Statistics 17.0 software package.

## Additional files

Additional file 1: Gene Ontology enrichment classifications of the differentially expressed genes up-regulated between the yellow-green and re-greening stages. (XLSX 14 kb) Additional file 2: Gene Ontology enrichment classifications of the differentially expressed genes down-regulated between the yellow-green and re-greening stages. (XLSX 15 kb) Additional file 3: Unigenes involved in carotenoid biosynthesis. (XLSX 11 kb) Additional file 4: Unigenes involved in chlorophyll biosynthesis. (XLSX 10 kb) Additional file 5: Unigenes involved in theanine biosynthesis. (XLSX 11 kb) Additional file 6: Primers used for quantitative reverse transcription polymerase chain reaction analyses. (XLSX 11 kb)

## Abbreviations

AIDA: Alanine decarboxylase
CAO: Geranylgeranyl diphosphate reductase
CLH: Chlorophyllase
DEG: Differentially expressed gene
DXS: 1-deoxyxylulose 5-phosphate synthase
FDR: False discovery rate
FPKM: Fragments per kilobase of exon per million mapped reads
G: Re-greening
GGPS: Geranylgeranyl diphosphate synthase
GO: Gene ontology
GOGAT: Glutamate synthase
GS: Glutamine synthetase
HCAR: Hydroxymethyl chlorophyll *a* reductase
IPP: Isopentenyl diphosphate
KEGG: Kyoto encyclopedia of genes and genomes
LCY-b: Lycopene β-cyclase
NCBI-nr: National Center for Biotechnology Information non-redundant
NOL: NYC1-like
NYC1: Chlorophyll *b* reductase
PPH: Pheophytinase
PSY: Phytoene synthase
qRT-PCR: Quantitative reverse transcription polymerase chain reaction
TAIR: The Arabidopsis Information Resource
TS: Theanine synthetase
UPLC–QqQ-MS: Ultra-performance liquid chromatography–triple quadrupole mass spectrometry
VDE: Violaxanthin deepoxidase
W: Albescent
YG: Yellow-green
Zep1: Zeaxanthin epoxidase
